# Transient stimulated Raman scattering spectroscopy and imaging

**DOI:** 10.1038/s41377-024-01412-6

**Published:** 2024-03-08

**Authors:** Qiaozhi Yu, Zhengjian Yao, Jiaqi Zhou, Wenhao Yu, Chenjie Zhuang, Yafeng Qi, Hanqing Xiong

**Affiliations:** 1 National Biomedical Imaging Center, College of Future Technology, Peking University, Beijing, 100871 China; 2 Biomedical Engineering Department, College of Future Technology, Peking University, Beijing, 100871 China

**Keywords:** Raman spectroscopy, Biophotonics

## Abstract

Stimulated Raman scattering (SRS) has been developed as an essential quantitative contrast for chemical imaging in recent years. However, while spectral lines near the natural linewidth limit can be routinely achieved by state-of-the-art spontaneous Raman microscopes, spectral broadening is inevitable for current mainstream SRS imaging methods. This is because those SRS signals are all measured in the frequency domain. There is a compromise between sensitivity and spectral resolution: as the nonlinear process benefits from pulsed excitations, the fundamental time-energy uncertainty limits the spectral resolution. Besides, the spectral range and acquisition speed are mutually restricted. Here we report transient stimulated Raman scattering (T-SRS), an alternative time-domain strategy that bypasses all these fundamental conjugations. T-SRS is achieved by quantum coherence manipulation: we encode the vibrational oscillations in the stimulated Raman loss (SRL) signal by femtosecond pulse-pair sequence excited vibrational wave packet interference. The Raman spectrum was then achieved by Fourier transform of the time-domain SRL signal. Since all Raman modes are impulsively and simultaneously excited, T-SRS features the natural-linewidth-limit spectral line shapes, laser-bandwidth-determined spectral range, and improved sensitivity. With ~150-fs laser pulses, we boost the sensitivity of typical Raman modes to the sub-mM level. With all-plane-mirror high-speed time-delay scanning, we further demonstrated hyperspectral SRS imaging of live-cell metabolism and high-density multiplexed imaging with the natural-linewidth-limit spectral resolution. T-SRS shall find valuable applications for advanced Raman imaging.

## Abstract (web summary)

A time-domain excitation strategy for stimulated Raman scattering was achieved by manipulating vibrational wave packet interference. The new method enables simultaneous sub-mM level sensitivity and natural-linewidth-limit Raman spectra.

## Graphical abstract

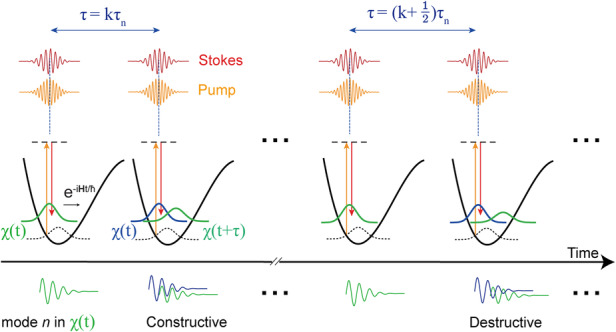

## Introduction

As an intrinsic contrast of the molecular structure, Raman scattering has long been used as an essential contrast to visualize molecular distribution and dynamics in a variety of systems. Harnessing the high gain of stimulated emission and its linear concentration dependence, stimulated Raman scattering (SRS) is rising as one of the most widely used Raman modalities for biomedical imaging in recent years^1–4^. At room temperature and in condensed phases, many vibrational modes predominantly feature a lifetime-broadened linewidth (i.e., the natural linewidth limit)^5^. However, fine spectral features approaching such a fundamental limit are beyond the resolution of SRS microscopy. This is because the current mainstream SRS imaging methods, whether excited by narrow-band picosecond-pulsed lasers^6,7^, broadband femtosecond-pulsed lasers^8^, or both^9,10^, are all based on frequency-domain excitation strategies. While short laser pulses are favored in nonlinear excitation, the time-energy uncertainty draws a fundamental limit on the frequency accuracy, resulting in inevitable spectral broadening. As a compromise, the spectral resolution of a few to tens of cm^−1^ is commonly adopted by current state-of-the-art SRS imaging systems for mM-level detection sensitivity^6–8,11,12^, causing observable spectral broadening for typical Raman modes (~10 cm^−1^ linewidth), especially for those triple-bond modes used for bioorthogonal Raman probes^11,13–15^. While the spectral broadening in frequency-domain SRS imaging has a minor effect on conventional label-free applications (in most cases, mapping the abundant overall proteins and lipids), it does cause difficulties for cutting-edge high-density bioorthogonal labeling^11,13^, super-multiplexing Raman imaging^14,15^, and advanced sensing^16–18^, in which high spectral resolution and sensitivity are equally critical. To further extend the applications of SRS imaging, a new hyperspectral excitation strategy that preserves the natural-linewidth-limit spectral lines with state-of-the-art sensitivity at the same time is highly demanded.

Though it is impossible in the frequency domain, breaking the conjugation between spectral resolution and pulse duration can be elegantly achieved in the time domain^19^. For instance, Fourier spectroscopy approaching the natural-linewidth-limit spectral lines can be achieved by measuring the free-induction decay of impulsive excitations, as demonstrated by impulsive stimulated Raman scattering (ISRS)^20–23^, Fourier-transform coherent anti-Stokes Raman scattering (FT-CARS)^24,25^, and other ultrafast vibrational spectroscopy methods^26–29^, etc. While a relatively high spectral resolution of low-wavenumber Raman modes can be achieved by ISRS, the underlying physical principle of ISRS signal formation (i.e., detecting the refractive index modulation induced by the molecular vibration) results in inevitable discrepancies with the spontaneous Raman spectra, and the single-band excitation strategy hinders its accessibility to the high-wavenumber bands (such as the C-H stretching band around 2900 cm^−1^, etc.) widely used in biomedical applications^22,23,30,31^. What’s more, even though ISRS is excited with temporally much shorter femtosecond pulses, comparable sensitivity to that of the frequency-domain methods has not been reported.

**Fig. 1:**
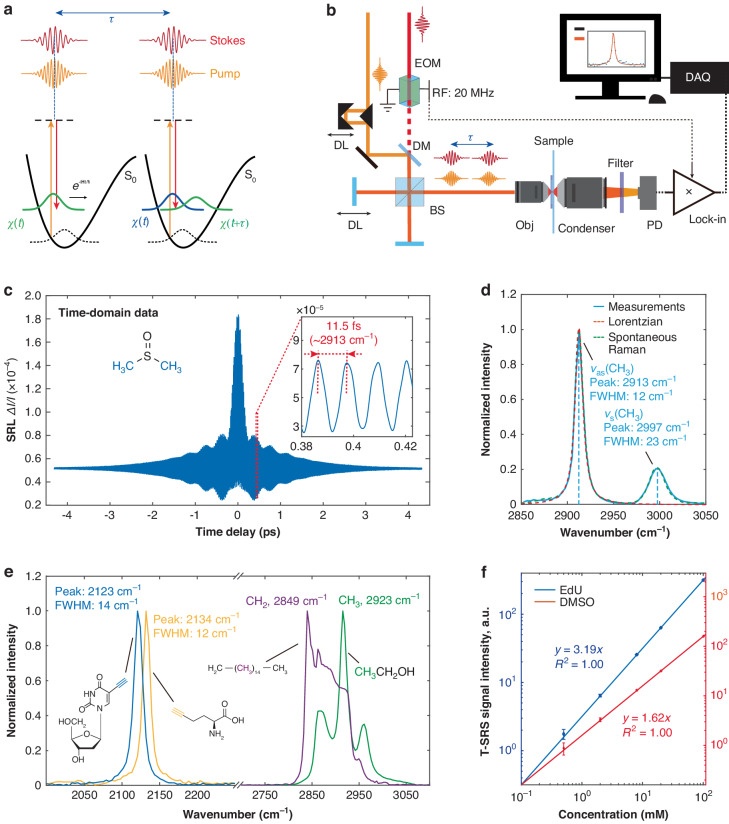
Transient stimulated Raman scattering (T-SRS). **a** Schematics and **b** setup of T-SRS spectroscopy. χ(t) and χ(t+τ) are the two vibrational wave packets excited by the two corresponding pump and Stokes pulse pairs, respectively. EOM for electro-optic modulator; DL for delay line; DM for dichroic mirror; BS for beam splitter; PD for photodiode; Obj for objective; DAQ for data acquisition card. **c** The time-domain stimulated Raman loss (SRL) signal of C-H stretching modes of dimethyl sulfoxide (DMSO) under T-SRS excitation and its (**d**) Fourier transform. Green dashed curve is the spontaneous Raman spectrum recorded with 0.65 cm^−1^ spectral resolution. **e** T-SRS spectra of the C-H stretching modes of hexadecane (purple) and ethanol (green), the alkyne-mode spectra of 100-mM L-homopropargylglycine (Hpg, yellow) and 5-ethynyl-2’-deoxyuridine (EdU, blue) in DMSO, respectively. **f** Concentration dependence of the T-SRS signal. Blue points for alkyne-mode of EdU; red points for the asymmetry CH_3_ stretching mode of DMSO. Error bar represents the standard deviation of 50 data points of the off-resonance region

Here we report a general time-domain SRS spectroscopy named transient stimulated Raman scattering (T-SRS) (Fig. 1). Instead of probing the nonlinear index modulation introduced by the propagation of a single vibrational wave packet (i.e., signal formation in ISRS), we generate vibrational wave packet interference by two successive SRS excitations with ideal two copies of the same femtosecond pulse pairs (i.e., the pump and Stokes pulses) separated by well-defined time delay (τ) (Fig. 1a, b). Scanning the time delay results in periodic oscillations between constructive and destructive interference with the eigenfrequencies of corresponding Raman modes, which modulates the vibrational state populations, and therefore encodes vibrational features on the time-domain signal trace of stimulated Raman loss (SRL) (or gain). Then, Raman spectra with linewidths limited only by the coherence length of the vibrational wave packet (i.e., the natural linewidth limit) can be obtained by Fourier transform of the time-domain SRL signal. T-SRS inherits the widely used heterodyne detection of SRL, which results in highly quantitative contrast (i.e., linear concentration dependency and low background interferences). And thanks to the high excitation efficiency of Fourier-transform-limit femtosecond pulses, T-SRS shows better sensitivity to its state-of-the-art frequency-domain counterparts. We boost the sensitivity of typical Raman modes to the sub-mM level with ~150-fs laser pulses. Further, by introducing an all-plane-mirror (i.e., dispersion-free and beam profile preserving) high-speed time-delay scanning strategy for T-SRS excitation, we demonstrated live-cell metabolic imaging and high-density multiplexed imaging with the natural-linewidth-limit spectral resolution. T-SRS is ready to go for advanced SRS microscopy.

## Results

### T-SRS spectroscopy

Figure 1b shows our setup for T-SRS spectroscopy. A femtosecond-pulsed Yb^3+^-doped fiber laser (~150-fs pulse duration, 100-MHz repetition rate) and its second harmonic pumped optical parametric oscillator (OPO) were used to serve as the synchronized Stokes beam and pump beam, respectively. The temporal synchronized and spatial overlapped pump and Stokes pulse pair was sent to a Michelson interferometer to generate two identical copies of itself, with the time delay between them controlled by a high-precision linear-motion stage. The conventional collinear excitation geometry and heterodyne SRL detection were used^6^, with the Stokes beam modulated at 20-MHz for shot-noise-limited sensitivity. We pre-compensated the dispersion of the laser pulses so that near Fourier-transform-limited pulse durations were achieved at the focus (Fig. S1).

To demonstrate the concept of T-SRS, we first performed [−4.5 ps, 4.5 ps] time-delay scanning for T-SRS excitation of the two CH_3_ stretching modes of dimethyl sulfoxide (DMSO) (pump and Stokes pulses centered at 789 nm and 1030 nm, respectively). As predicted, strong oscillations matching the target vibrational eigenfrequencies and carrying a beating envelope were observed on the SRL signal trace (Fig. 1c), indicating wave packet interference of two adjacent vibrational modes. The decay of the beating envelope is ~2 times faster than that of the overall oscillations, suggesting a ~2 times difference between the linewidths of the two modes. This was further confirmed by the Fourier transform: two Lorentzian-like spectral lines centered at the corresponding resonance frequencies (i.e., 2913 cm^−1^ and 2997 cm^−1^, respectively) with the predicted linewidth difference (i.e., 12 cm^−1^ for asymmetric stretching versus 23 cm^−1^ for symmetric stretching) were observed, and the spectra are strictly identical to those measured by grating-based spontaneous Raman spectrometer with 0.65 cm^−1^ spectral resolution (Fig. 1d, Fig. S2), confirming the dominantly lifetime-broadened linewidths (i.e., the natural linewidth limit).

**Fig. 2:**
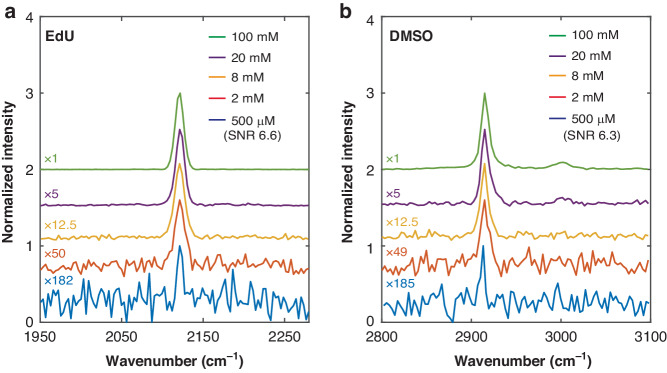
Sensitivity of T-SRS. The **a** nitrile mode of EdU and **b** the asymmetric CH_3_ stretching mode of DMSO were measured. The normalized spectra are vertically offset for clarity

To demonstrate the generality of T-SRS, we further performed T-SRS measurements of the C-H stretching modes of hexadecane and ethanol, the widely used alkyne tags of L-homopropargylglycine (Hpg)^32^ and 5-ethynyl-2’-deoxyuridine (EdU)^33^ (Fig. 1e), etc. Again, the T-SRS spectra are identical to those measured by high-resolution spontaneous Raman spectrometers^11^ (Fig. S3a). Furthermore, inheriting the classical heterodyne SRL detection, the T-SRS signal shows a strictly linear relation to the concentration (Fig. 1f). What’s more, compared to the well-known mM-level sensitivity of the current state-of-the-art SRS and CARS systems^6,34^, higher spectral sensitivity was achieved for typical Raman modes: Signal-to-noise ratios of 6.6 for the alkyne mode of 500-μM EdU and 6.3 for the asymmetry CH_3_ stretching mode of 500-μM DMSO (Fig. 2, Fig. S5) were achieved with millisecond-level dwell time under biocompatible laser intensity (details in “Materials and methods”), respectively. To our knowledge, without any enhancements from large conjugation systems^15^ and special electronic-resonance^14^, these are the first SRS spectra acquired with sub-mM concentration.

It should be noted that non-resonant backgrounds (i.e., such as four-wave mixing and multiphoton absorptions of impurities^35^, etc.) found in the conventional frequency-domain SRS spectroscopy can also be observed in T-SRS spectra (Figs. S4, S5). Since the coherences of the non-resonant backgrounds decay much faster than that of the Raman process, non-resonant backgrounds in the time domain can be easily solved by a time-gating trick. Basically, by removing the time-domain data section that corresponds to the overlap of the two excitation pulse pairs, non-resonant backgrounds in the T-SRS spectrum can be efficiently removed (Fig. S4, details in SI). Besides this time-gating trick, we also found that the overall non-resonant background has a line shape proportional to the excitation efficiency curve (i.e., the correlation function between the pump spectrum and the Stokes spectrum), and can be efficiently subtracted by a simple fitting model (details in SI).

### All-plane-mirror high-speed time-delay scanning

To harness the natural-linewidth-limit spectral lines for hyperspectral SRS imaging, it is important to achieve fast time-delay scanning for T-SRS excitation. Here we report an all-plane-mirror (i.e., no transmitted optics and curved surfaces) high-speed delay scanning method. The elegance of the all-plane-mirror design is that it introduces negligible dispersions and optical aberrations to the excitation pulse pairs, which guarantees ignorable phase errors in T-SRS excitation, and as a result, preserves the natural-linewidth-limit spectral lines (details in SI, Fig. S6). This unique feature clearly distinguishes our method from those advanced delay-scanning methods previously developed for vibrational spectroscopy and imaging^25,36–38^.

**Fig. 3:**
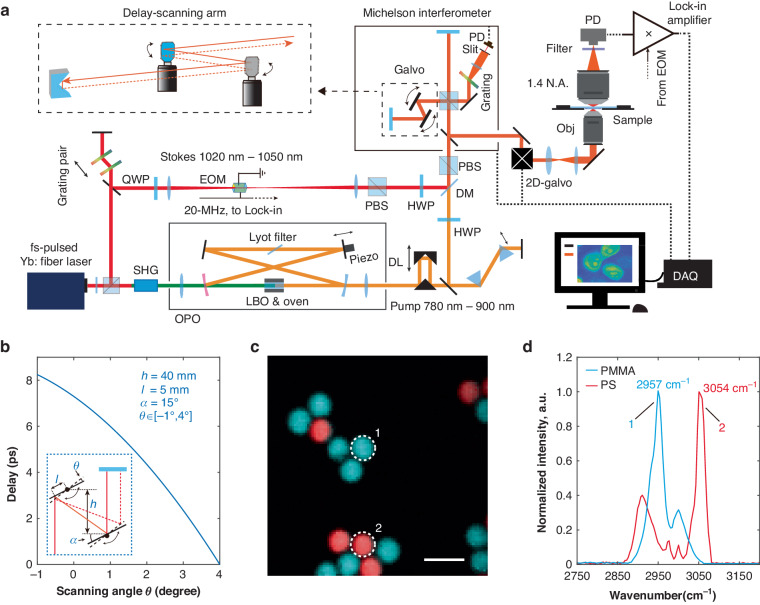
T-SRS microscopy. **a** The setup. SHG for second harmonic generation; OPO for optical parametric oscillator; LBO for lithium triborate; QWP and HWP for quarter and half wave plates; PBS and BS for polarized and unpolarized beam splitters; Galvo for galvanometer mirror. **b** A typical delay scanning configuration (inset) and its delay as a function of the galvanometer rotation angle. **c** Typical hyperspectral T-SRS imaging of polymethyl methacrylate (PMMA) and polystyrene (PS) beads and **d** the corresponding T-SRS spectra of the C-H stretching band. Scale bar: 10 μm

As shown in the dashed box of Fig. 3a, the time-delay scanning is achieved by the rotation of two synchronized parallel-aligned galvanometer mirrors. A retroreflector is used to shift the beam along the rotation axis so that the beam can be picked out after descanning. Figure 3b shows the scanning range of a typical setup, when the collimated laser pulses are input to the first mirror with 5-mm off-axis shift and a 15-degree incident angle, and the two mirrors are placed with 40-mm distance along the incident direction, rotating the mirrors in the [−1, 4]-degree angle range results in more than 8-ps range of delay scanning (detailed derivation in SI). The T-SRS spectra collected with such an ideal delay line show neglectable differences with the natural linewidth limit spectra measured by spontaneous Raman scattering (Fig. S6c). With such superb spectral resolution, nitrile modes of isotopologues with merely 26-cm^−1^ intervals can be easily distinguished with a scan of the delay line (Fig. S7). The response speed of our galvanometer mirrors enables a delay scanning rate of up to 1000 Hz. When equipped with much faster resonance mirrors, the delay scanning rate can be further improved by orders of magnitude^37^.

Upgrading the setup with the all-plane-mirror high-speed time-delay scanning and synchronizing T-SRS excitation with a standard laser scanning microscope (Fig. 3a), we performed hyperspectral SRS imaging. As the first demonstration, the mixture of 5-μm-diameter polymethyl methacrylate (PMMA) and polystyrene (PS) beads was captured in C-H stretching band. With ~21 mW pump power and ~11 mW stokes power after condenser, high contrast (SNR > 280) hyperspectral image was captured with millisecond-level spectral integration time (Fig. 3c), with the particles unambiguously distinguished by their corresponding Raman spectra (Fig. 3d). And to demonstrate the performance of T-SRS in the Raman fingerprint region, we further performed T-SRS imaging of the beads in the [950 cm^−1^, 1650 cm^−1^] spectral range (Fig. S8). Again, high SNR spectra and images were achieved. Notably, the Raman spectrum of the phenyl ring breathing mode (i.e., the peak of the red curve at 1001 cm^−1^) of PS was captured with ~6 cm^−1^ Raman linewidth, further confirming the high spectral resolution of T-SRS. Our current laser bandwidths (i.e., ~80 cm^−1^ and ~100 cm^−1^ bandwidths for pump and Stokes pulses, respectively) enable us to capture the Raman spectrum of ~124 cm^−1^ range (Fig. S7). To cover the [950 cm^−1^, 1650 cm^−1^] spectral range, we tuned the central wavelength of the pump pulse for 6 times and stitched the spectra. With temporally narrower excitation pulses (i.e., broader laser linewidths), a larger spectral range could be achieved.

### Hyperspectral live-cell SRS microscopy via T-SRS excitation

**Fig. 4:**
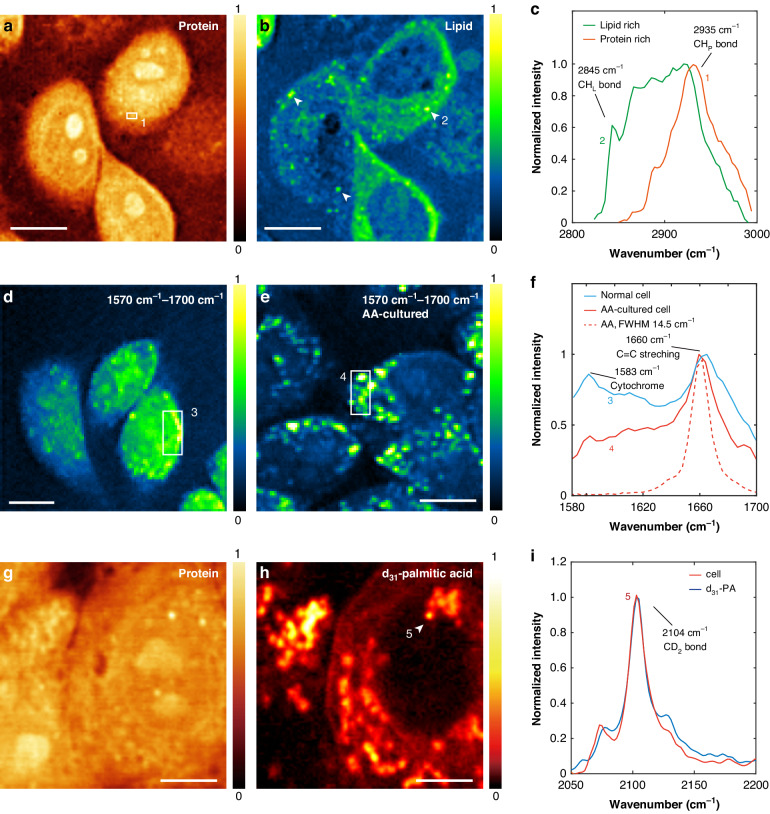
T-SRS-based live-cell chemical imaging. Label-free imaging of **a** proteins and **b** lipids of live cells. These two channels are unmixed from the overall hyperspectral data with the standard linear decomposition method^39^. **c** Typical T-SRS spectra of proteins (region 1 in (**a**), red curve) and lipids (region 2 in (**b**), Green curve). **d** Typical T-SRS imaging of normal *Hela* cell and **e** 50-µM arachidonic acid (AA) cultured *Hela* cell in the [1570 cm^−1^, 1700 cm^−1^] range. **f** shows the spectra of the marked regions in (**d**) and (**e**), respectively. The red dashed curve is the C=C stretching mode Raman spectrum of pure AA. **g** Protein channel reference and **h** The C-D stretching band of d_31_-palmitic acid labeled cells. **i** T-SRS spectrum of the marked structure in (**h**). Scale bar: 10 μm for (**a**), (**b**), (**d**) and (**e**); 5 μm for (**g**) and (**h**)

To demonstrate T-SRS as a general tool for live-cell chemical imaging, we performed the typical applications of conventional frequency-domain SRS by T-SRS. With a moderate 21-mW and 11-mW pump and Stokes power, we first performed the widely used label-free imaging of overall proteins and lipids in live cells (Fig. 4a–c). As shown in Fig. 4a and Fig. 4b, the protein component and lipid component can be efficiently unmixed from the T-SRS hyperspectral data set, with the typical spectrum of the protein-rich (or lipid-rich) region well matches the benchmark results^7,8,39^ (Fig. 4c). Furthermore, live-cell imaging in the fingerprint region can also be routinely achieved (Fig. 4d–f), T-SRS excitation of the [1570 cm^−1^, 1700 cm^−1^] spectral range clearly revealed Raman features of the C=C stretching (i.e., the peak at 1660 cm^−1^) and the cytochrome C (i.e., the peak at 1583 cm^−1^)^40^. Interestingly, cell up-taking of unsaturated fatty acid, such as arachidonic acid (AA), can be visualized by the C=C stretching signal (Fig. 4d–f).

To show the feasibility of T-SRS for bioorthogonal Raman probes, we further performed live-cell lipid metabolic imaging with d_31_-palmitic acid labeling^41^. Such a stable isotope-edited fatty acid enables the tracking of lipid uptake and metabolism in the cell-silence region^42^. After the cells were cultured in 50-μM d_31_-palmitic acid for 6 h, high SNR spectra of C-D stretching bands were detected (Fig. 4g-i), with the C-D signal mainly accumulated in the lipid droplets (Fig. 4h). Notable differences between the C-D band spectra of live cells and the d_31_-palmitic acid solution were revealed (Fig. 4i), which can be used to track the metabolic process of palmitic acid. These results are again consistent with the benchmark results^41,43^.

The feasibility of T-SRS with both label-free and bioorthogonal live-cell chemical imaging enables the seamless applications of T-SRS in biomedicine. Notably, larger spectral range and higher sensitivity may be achieved with temporally shorter laser pulses (i.e., broader bandwidths and higher peak intensities), which may bring in new opportunities for biomedical discoveries.

### T-SRS based high-density barcoding

**Fig. 5:**
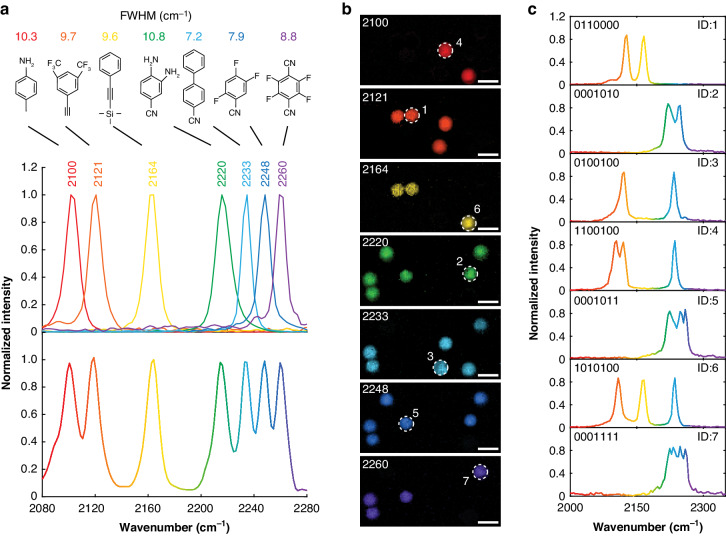
T-SRS-based barcoding. **a** Seven small-molecule probes and their corresponding T-SRS spectra in the triple-bond band. The upper panel shows the separated spectrum of each probe, the bottom panel shows the overall spectrum of the solution with seven probes mixed. **b** Signal of the encoded PMMA beads in each probe channel. **c** Spectra of typical encoded PMMA beads in the triple-bond band labeled in (**b**). Scale bar in (**b**): 10 μm

Finally, to release the advantage of the superb spectral resolution, we performed high-density barcoding with multiplexing probes spectrally spaced as fine as ~12 cm^−1^ (Fig. 5). In comparison, conventional SRS imaging uses probes with more than 20 cm^−1^ spacing due to the limited spectral resolution^14,15^. As shown in Fig. 5a, seven commercial-available small molecules are selected as the model barcoding probes. Under the natural-linewidth-limit resolving of T-SRS, many benzonitrile and ethynylbenzene derivatives reveal less than 10 cm^−1^ triple-bond Raman linewidths (Fig. S3b). These narrow linewidths suggest that high-density multiplexing and barcoding with Raman probes separated by ~10 cm^−1^ can be achieved by T-SRS. As a demonstration, we barcoded PMMA beads with these model probes by the widely used swelling-diffusion technique^15,44,45^ (details in “Materials and methods” and SI). T-SRS enables the decoding of these high-density barcodes with single-round excitation. As shown in Fig. 5b, signals of the seven probes can be routinely unmixed from the hyperspectral T-SRS imaging. Although embedding the probes in PMMA beads slightly broadened the Raman linewidths, the overall T-SRS spectra of the beads in the triple-bond band unambiguously show the characteristic peaks (Fig. 5c). Chemical engineering Raman modes with even narrower linewidths (i.e., longer lifetimes) should enable much higher-density multiplexing.

With the current binary digits (i.e., ‘0’ and ‘1’) encoding (Fig. 5c), the 7 probes enable the encoding of up to 2^7^ = 128 different targets. By slightly increasing the number of probes or digits, thousands of different codes can be generated^15^, which is far beyond the multiplexity of the cutting-edge inorganic particles^46^ and microlasers^47^. Given the well-established conjugations of biomarkers (such as antibodies, nucleic acid fragments, etc.) to polymer beads in the biomedical industry^48–51^, T-SRS-based barcoding could initiate high-throughput biomedical screening and diagnosis techniques.

## Discussion

As a summary, we demonstrated T-SRS (Figs. 1 and 2), an ultrafast time-domain SRS spectroscopy that can be used as a general contrast for chemical imaging (Figs. 3–5). Comparing to conventional time-domain SRS techniques^20–22^, T-SRS features the heterodyne SRL (or SRG) signal formation of modern SRS imaging^6^, which guarantees shot-noise-limited sensitivity, highly interpretable and quantitative contrast, and imaging-friendly collinear excitation geometry. As a complementary to the widely used frequency-domain SRS techniques, T-SRS features natural-linewidth-limit spectral line shapes, intrinsically hyperspectral readout, and higher sensitivity (Figs. 1 and 2, Fig. S5), which makes it an ideal solution for fast-dynamic chemical sensing and high-density multiplexed imaging. To release the power of T-SRS on chemical imaging, we introduced an all-plane-mirror time-delay scanning strategy (Fig. 3). Distinguished from the established delay scanning strategies^25,36–38^, this all-plane-mirror delay line enables high-throughput spectra acquisition with neglectable phase error, supporting T-SRS imaging as a valuable SRS modality for applications where high spectral resolution is critical (Fig. 5). The dispersion-free and aberration-free features of our novel delay scanning strategy could also find applications in other ultrafast spectroscopy^52,53^.

Our current T-SRS system enables the spectral acquisition of up to a few milliseconds per spectrum, which corresponds to hyperspectral imaging of a few minutes per frame. This is relatively slow when compared with the state-of-the-art Frequency-domain methods^7,8,10,37^. However, this does not mean T-SRS intrinsically has lower throughput. First, higher speed resonance mirrors can be used to significantly increase the delay scanning (i.e., spectral acquisition) to microsecond level^37^. The second and the most important point, while the spectral range of frequency-domain methods is inversely proportional to the speed of spectral acquisition, the spectral range of T-SRS imaging is only determined by the laser pulses bandwidths because of simultaneous and impulsive excitation of Raman modes. T-SRS can easily boost the spectral range without increasing the acquisition time. Limited by the bandwidths of our excitation laser pulses, the FWHM spectral range of our T-SRS system is ~124 cm^−1^ (Fig. S7). Excited with shorter laser pulses (i.e., broader bandwidths), such as few-cycle pulses^25^, T-SRS has the potential to reveal the panoramic view of investigated systems without tuning the laser (i.e., ideally mimics the full-range spontaneous Raman spectra with SRS excitation efficiency).

Except for the extended spectral range, shorter pulses intrinsically support higher sensitivity (or higher imaging speed on the other hand) for nonlinear contrast like SRS. This point is actually supported by our current results: excited by merely ~12-mW and ~40-mW of femtosecond-pulsed pump and Stokes power, T-SRS boosts the sensitivity to sub-mM level (Fig. 2), beyond the mM-level sensitivity of the state-of-the-art Frequency-domain SRS systems^6,8^ excited by picosecond-pulsed lasers of much higher average power. So, T-SRS imaging with higher sensitivity and throughput may be anticipated with further improvements of laser source.

Furthermore, unlike conventional SRS imaging techniques^7,8,11,12^, T-SRS prefers transform-limited ultrashort femtosecond laser pulses for excitation, suggesting higher compatibility with other nonlinear optical imaging modalities, such as multiphoton-excited fluorescence, second harmonic generation, ultrafast pump-probe techniques, etc. Combining T-SRS with those modalities shall open new opportunities for biomedical findings or even broader scenarios.

## Materials and methods

### System construction

A home-built Yb^3+^-doped femtosecond fiber laser (100 MHz repetition rate, 1030-nm center wavelength, 15-nm FWHM bandwidth) and its SHG-pumped OPO system were used to prepare the synchronized Stokes and pump pulse, respectively. With dispersion compensation, the pump pulse duration was typically ~150 fs, the Stokes was ~140 fs, all pulses were close to the Fourier-transform limit. For the two outputs of the interferometer, one output was coupled into a confocal microscope (IX73, Olympus) for T-SRS excitation; the other one is spectrally spread by a piece of high-line-density grating, and a tiny portion (less than 0.1 nm) of the spread spectrum was sent to a photodiode to generate the reference signal for time-delay calibration. All T-SRS experiments are performed with the same objective (UPLSAPO, 60×, water immersion, 1.2 NA, Olympus). The lateral resolution of our system is tested to be ~450 nm. For SRL signal detection, the pump beam was collected by a high NA oil-immersion condenser (U-TLO, 1.4NA, Olympus), separated by a bandpass filter (BBP700-950A, Rayan), and further sent to the detector (s3590-09, Hamamatsu). A high-speed Lock-in amplifier (HF2LI, Zurich Instrument) was used for signal demodulation. The Lock-in amplifier worked in the internal reference mode. The reference RF signal (20 MHz, 250 mV Vpp) was amplified by a power amplifier (ZX60-100VH+, Mini-circuits) to drive the resonance electro-optic modulator (EO-AM-R-20-C1, Thorlabs) for Stokes beam modulation. A Multifunction I/O card (USB-6363, NI) driven by a home-written LabVIEW program was used to synchronize the time-delay scanning, imaging scanning, and SRL signal & calibration signal acquisition.

### Sample preparation

For solution sample preparation, the solution was filled in the chamber of an imaging spacer (GBL654006, sigma) sandwiched by the standard microscope coverslip (CG15NH1, Thorlabs) and slide. The probe molecules (ACS NO. 14235-81-5, 88444-81-9, 2170-06-1, 17626-40-3, 2920-38-9, 98349-22-5, and 1835-49-0, Konoscience), and the solvent Dimethyl sulfoxide (D8418, Sigma) are directly used without further purification.

Protocols of cell sample preparation can be found in previous research^43^. For barcoded beads preparation, protocols are slightly modified from previous reports^15,44^. Selected benzonitrile and ethynylbenzene derivatives were dissolved in DMSO with concentrations listed in Supplementary Table 2 to form the probe solutions. Meanwhile, the PMMA beads solution (about 10% in deionized water) was mixed with the Pluronic F-127 (surfactant, 20% in DMSO) solution with 19:1 volumetric ratio to prepare the beads solution. Then, 20-μL probe solution for each barcode were mixed with 30-μL tetrahydrofuran (THF) and 10-μL beads solution. The mixtures were vortexed for 3 min and shaken for 2 h at room temperature to generate barcoded beads with high homogeneity. At last, the barcoded beads were separated from the solutions by 2-min 8000-rpm centrifugation and washed with 200-μL deionized water. For sample preparation, the barcoded beads were mixed and spread on a coverslip. Meanwhile, 8 μL hot agarose solution (6% in deionized water, 80 °C) was dripped in the well of the imaging spacer (GBL654006, sigma) attached on a glass slide. The surface of the coverslip with beads spread on it was quickly attached to the hot agarose, sealed the chamber created by the imaging spacer. The sample was ready after the agarose was cooled down.

### Data acquisition

All the spontaneous Raman spectra were measured by a commercial Raman spectrometer (LabRAM HR Evolution, Horiba) with the spectral resolution set to 0.65 cm^−1^. For all the T-SRS measurements, the delay scanning range was set to larger than 8 ps to ensure an instrument spectral resolution higher than 5 cm^−1^ (i.e., $\frac{1.2}{8{\rm{ps}}}$ for the box-car apodization function). For T-SRS spectra shown in Figs. 1 and 2, Fig. S3 and Fig. 5a, the sample rate was set to 400 kHz, and ~30,000 points of the SRL signal and the time-delay calibration signal were sampled synchronously for every round of delay scanning, corresponding to ~8-ps delay range. Details of the data acquisition for T-SRS are discussed in SI. For T-SRS imaging shown in Figs. 3–5, the sample rate was set to 1 MHz. And for each pixel, we performed one round of delay scanning (both clockwise rotation and counterclockwise rotation of the galvanometers were used for delay scanning), and ~30,000 points (corresponding to ~8 ps delay range) were used for spectral reconstruction, the pixel dwell time is therefore ~30 ms. Typical imaging dimension is 100 × 100 pixels, the overall acquisition time of one hyperspectral imaging stack is ~6 minutes. The spectra are the amplitudes of the fast Fourier transformations of corresponding time-domain SRL signal. For Fig. 1f and Fig. 2, the powers of pump and Stokes beams were measured as 12 mW and 40 mW after the condenser, respectively. For all other T-SRS excitations, the powers of pump and Stokes beams were measured as 21 mW and 11 mW after the condenser, respectively. For spectra of low concentrations, non-resonant backgrounds from four-wave mixing and multiphoton absorptions of impurities become obvious. The spectra shown in Fig. 2 are all pure SRS spectra. Their backgrounds were removed by subtracting fitted curves proportional to the excitation efficiency curve (details in SI).

### Supplementary information

Supplementary Information for Transient stimulated Raman scattering spectroscopy and imaging

## Data Availability

The data and code used in this research are available from the corresponding author upon reasonable request.
