# Supplementary material for: Transient stimulated Raman scattering spectroscopy and imaging
Source: Light Sci Appl. 2024 Mar 8;13:70. doi: 10.1038/s41377-024-01412-6 (PMC10920877; doi:10.1038/s41377-024-01412-6)
Supplement: Supplementary file 1 — Supplementary Information for Transient stimulated Raman scattering spectroscopy and imaging [file 41377_2024_1412_MOESM1_ESM.pdf]

Supplementary Information for

Transient stimulated Raman scattering spectroscopy and imaging

Qiaozhi Yu[1,3], Zhengjian Yao[1,3], Jiaqi Zhou[1], Wenhao Yu[2], Chenjie Zhuang[2], Yafeng Qi[2], Hanqing Xiong[1,*]

[1]National Biomedical Imaging Center, College of Future Technology, Peking University, Beijing 100871, China

[2]Biomedical Engineering Department, College of Future Technology, Peking University, Beijing 100871, China

[3]These authors contributed equally.

* Corresponding author. E-mail: xiong.hanqing@pku.edu.cn

This file contains the following contents:

Figure S1-S9

Non-resonant background subtraction for T-SRS

All-plane-mirror high-speed time-delay scanning

Details of data acquisitions

Supplementary Table 1

Supplementary Table 2

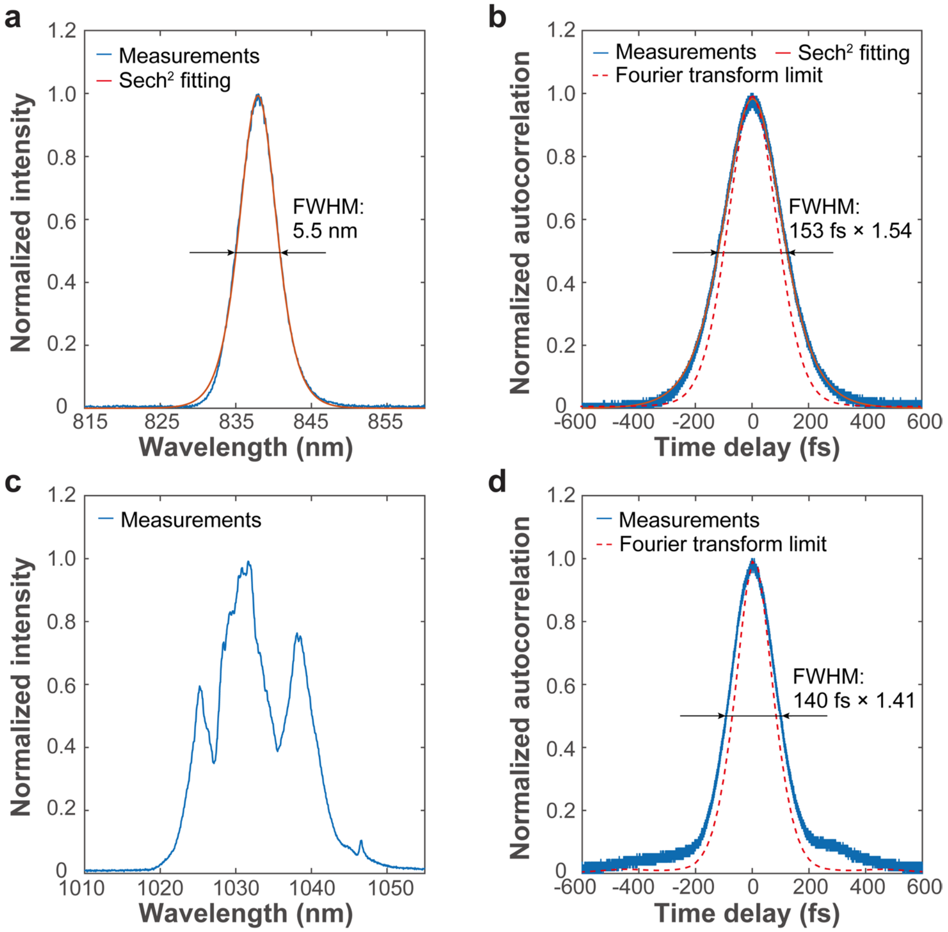

Fig. S1 Characterization of the excitation pulses. (a) and (b) show typical spectrum and autocorrelation of the pump pulse. (c) and (d) show the spectrum and autocorrelation of the Stokes pulse. Each pulse was compensated close to its corresponding Fourier transform limit (red dashed curves in (b) and (d)), separately.

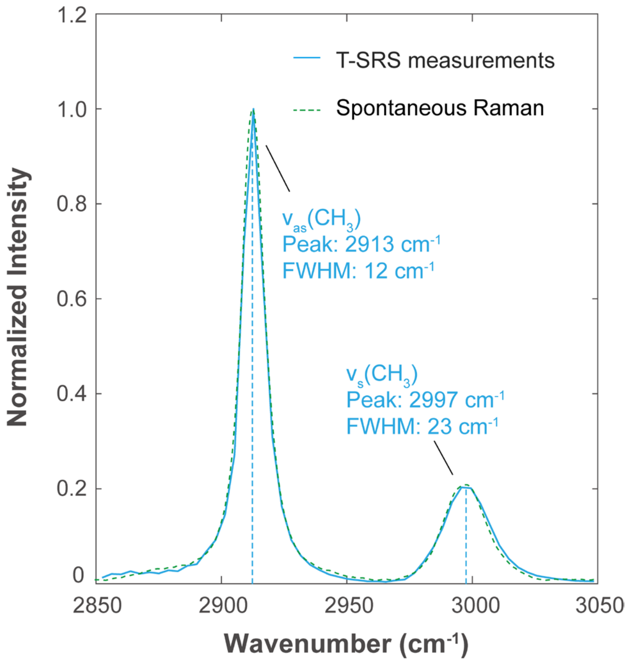

Fig. S2 Comparison between the spontaneous Raman spectra (0.65 cm^{-1} spectral resolution) and the T-SRS spectra of C-H stretching mode of DMSO. This figure is the enlarge of the **Fig.1d**.

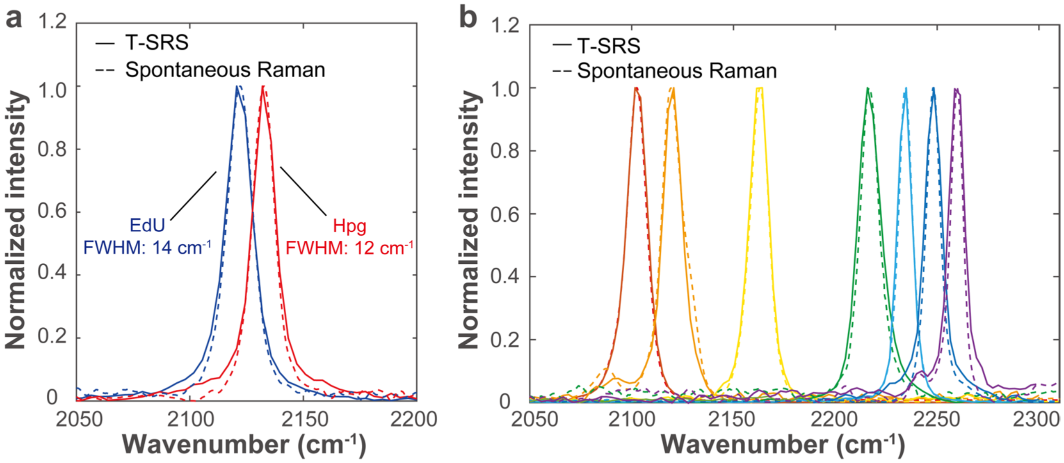

Fig. S3 Comparison between the spontaneous Raman spectra (0.65 cm^{-1} spectral resolution) and the T-SRS spectra of triple-bond modes. Chemical structures of the corresponding molecules in (a) can be found in **Fig.1e**, Chemical structures of the corresponding molecules in (b) can be found in **Fig.5a**.

Non-resonant background subtraction for T-SRS

For T-SRS spectra of low concentration molecules (i.e., usually less than 10 mM), the non-resonant backgrounds from four-wave mixing and multiphoton absorptions of impurities become obvious. As an extreme case, the raw T-SRS spectrum of 500-μM EdU in DMSO (**Fig.S5**) shows an overall non-resonant background ~9 times of the pure T-SRS signal of the C≡C mode. Similar non-resonant background has also been observed by conventional frequency-domain SRS techniques[1]. Usually, frequency modulation is used to suppress the non-resonant background[2-4]. Here we provide two strategies to remove the the non-resonant backgrounds for T-SRS spectroscopy.

For the first strategy, the physics behind the background subtraction is that those non-resonant backgrounds are almost all instantaneous processes whose lifetimes can be neglected compared with that of a typical vibrational excited state. In T-SRS, these backgrounds can be coupled into the Raman band only when the two pulse pairs overlap. As long as the two pulse pairs are separated, the coherence of the backgrounds excited by the first pulse pair will quickly die out and cannot interact with the coherence excited by the second pulse pair. So, the non-resonant background of T-SRS can be easily removed in the time domain by a virtual time-gating process (i.e., removing the data section in the time-delay window when the two pulse pairs overlap, see **Fig.S4a,c**) after the data has been recorded. This time-gating process can be done for both double-side delay-scanning data trace (**Fig.S4a**) and single-side delay-scanning data trace (**Fig.S4c**). **However, the time-gating points must be carefully selected to avoid spectral distortion (caution!).** For Fourier transform spectroscopy[5], if double-side delay-scanning data trace is used, the spectrum is the absolute value of the Fourier transform (**Fig.S4a,b**). In this case, to avoid spectrum distortion, the two time-gating points must be selected so that the end points have the same phase of vibrational coherence. If the single-side delay-scanning data trace is used, the spectrum is the real part of the Fourier transform[5] (**Fig.S4c,d**). In this case, to avoid spectrum distortion, the time-gating point must be selected so that the phase of the vibrational coherence is zero, otherwise the real part and the imaginary part of the Fourier transform will be mixed up. Luckily, in our experience, the time-gating point is the same for T-SRS excitation with the same time-delay-scanning configuration. Once the time gating points are found, it works for all the data collected with the same time-delay scanning configuration.

The second strategy is based on our observations that the overall non-resonant background matches very well with the excitation efficiency curve of the system. For the excitation efficiency curve $E(\Omega_R)$, we mean the correlation function between the pump-pulse spectrum and the Stoke-pulse spectrum

$$E(\Omega_R) \propto \left| \int E_{pump}(\omega) E_{Stokes}(\omega - \Omega_R) d\omega \right|^2. \quad (1)$$

Here Ω_R is the Raman shift, $E_{pump}(\omega)$ and $E_{Stokes}(\omega)$ are the square roots of the power spectral densities of the pump pulse and Stoke pulse, respectively. Note that this excitation efficiency estimation will be accurate only if the pulses are very close to the Fourier transform limit. By fitting the overall non-resonant background by the excitation efficiency curve $E(\Omega_R)$, one can efficiently remove the non-resonant background. **Fig.S5** show one of the extreme cases, an overall non-resonant background ~9 times of the pure T-SRS signal can be successfully removed.

The first strategy (i.e., time-gating) is physically elegant. But time-gating will result in slightly signal attenuation, and the gating points must be carefully selected. The second strategy (i.e., excitation efficiency curve fitting) features high stability and minimum signal attenuation. In the manuscript, we use the second strategy for all the subtractions of non-resonant backgrounds.

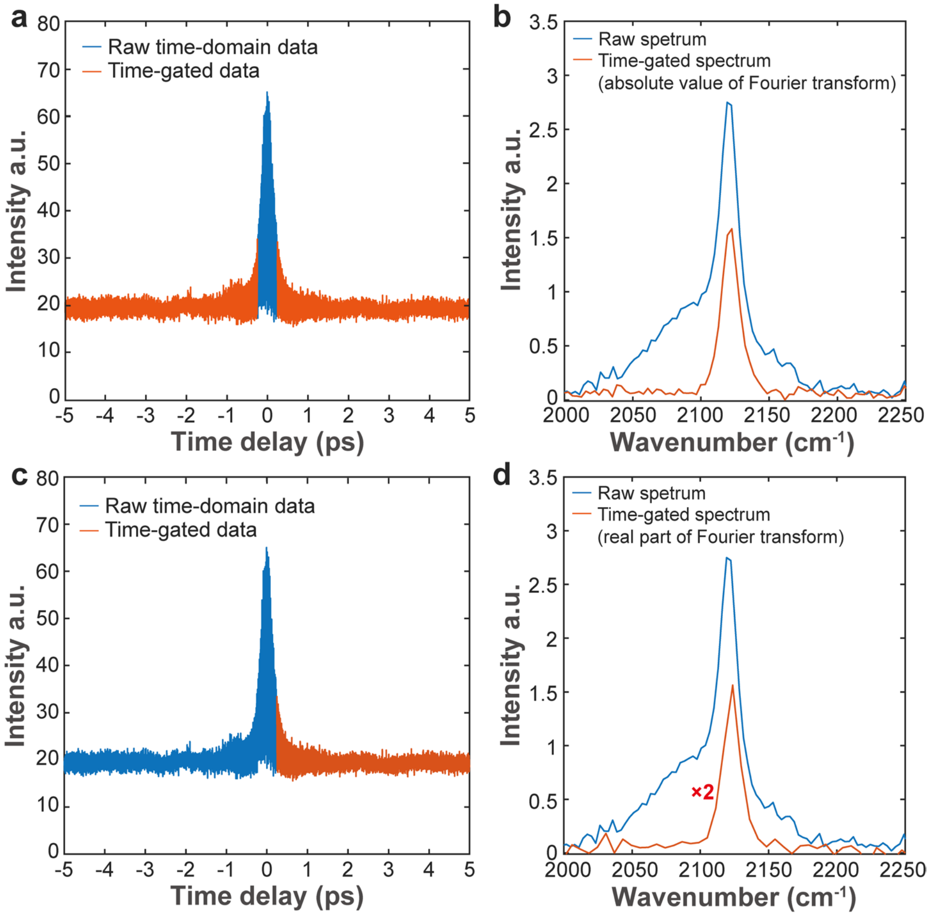

Fig. S4 Removing the non-resonant background by time gating. (a) and (b) show the time gating traces for double-side and single-side time delay scanning data, respectively. Their corresponding Fourier transforms are shown in (b) and (d), respectively. The data is recorded from the 8-mM EdU in DMSO. Note that the spectra in (b) are the absolute value of the corresponding Fourier transforms, the red curve in (d) is the real part of the corresponding Fourier transform.

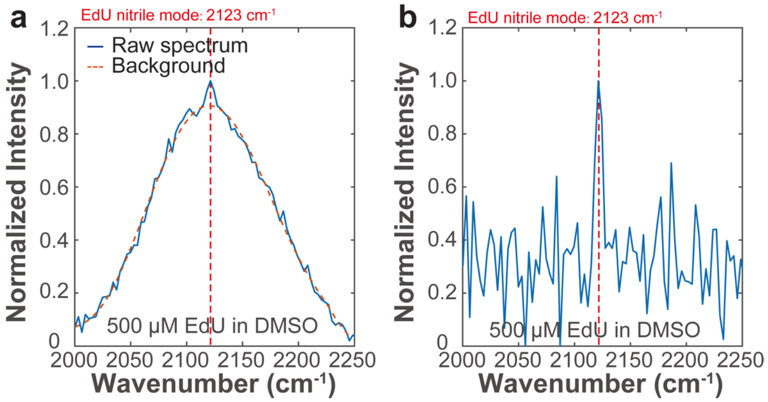

Fig. S5 Subtraction of non-resonant background by excitation efficiency curve fitting. (a) Backgrounds induced by four-wave mixing and multiphoton absorptions of impurities show an overall line shape proportional to the excitation efficiency of the system (i.e., the correlation function of the pump spectrum and the Stokes spectrum, the dashed red curve), and can be removed to reveal (b) the pure SRS spectrum. Here the raw data (blue curve in (a)) is recorded from the 500-μM EdU in DMSO.

All-plane-mirror high-speed time-delay scanning

A pair of parallel-aligned standard galvanometer mirrors (S-9210, Sunny Technology) were used for delay scanning. The geometry of the setup can be found in **Fig.S6a**. Based on the coordinates and labeling shown in **Fig.S6a**, the coordinates of *B'*, *C*, and *C'* can be derived by basic analytical geometry.

$$B'_x = 0\,, \tag{1}$$

$$B'_y = l\sin(\alpha) + m - l\cos(\alpha)\tan(\alpha+\theta)\,, \tag{2}$$

$$C_x = [h - l\sin(\alpha)]\tan(2\alpha)\,, \tag{3}$$

$$C_y = m - h + l\sin(\alpha)\,, \tag{4}$$

$$C'_x = \frac{[h + a\tan(\alpha+\theta)]\{[h - l\sin(\alpha)]\tan(2\alpha) - l\cos(\alpha)\}}{\tan(\alpha+\theta) + \tan\left(\frac{\pi}{2} - 2\alpha - 2\theta\right)}\,, \tag{5}$$

$$C'_y = -C'_x\tan\left(\frac{\pi}{2} - 2\alpha - 2\theta\right) + l\sin(\alpha) + m - l\cos(\alpha)\tan(\alpha+\theta)\,. \tag{6}$$

The optical path can then be calculated based on equations (1)-(6) as

$$S = B'_y + \sqrt{[C'^2_x + (C'_y - B'_y)^2]} + D_y - C'_y\,. \tag{7}$$

Then the relative time delay is

$$delay = 2\frac{S - \min(S)}{c}\,. \tag{8}$$

Here c is the speed of light in vacuum. And the drift on the second mirror (off the rotation axis) (namely CC') is

$$d_{off} = \sqrt{[(C'_x - C_x)^2 + (C'_y - C_y)^2]}\,. \tag{9}$$

Set α = 15°, l = 5 mm, h = 40 mm, suppose θ scanning from -1° to 4°, the scanning range is larger than 8 ps, as shown in **Fig.3b**. Note that d_{off} should not exceed the half-width of the second mirror.

Based on the calculation above, linear delay increments could be generated by customizing the driving voltage (which is linear to the rotation angle) of the galvanometer mirrors when performing delay scanning (**Fig.3b**). As shown in **Fig.S6b,c**, the measurement of the C≡C mode of EdU by this strategy proved that accurate linear delay scanning is achieved since natural-linewidth-limit spectral line can be acquired by direct one-step Fourier transform of the time-domain SRL signal.

To guarantee high accuracy under electronic noise perturbation, the absolute time delay of every sample point can be further calibrated by the interference trace of one arm of the interferometer recorded synchronously (see **Fig.3a**).

This design contains no transmitted optics and curved surfaces, which means that the delay line introduces no dispersions and no aberrations. These features make it an ideal delay line for handling ultrafast pulses, which should find valuable applications for a wide variety of ultrafast spectroscopy.

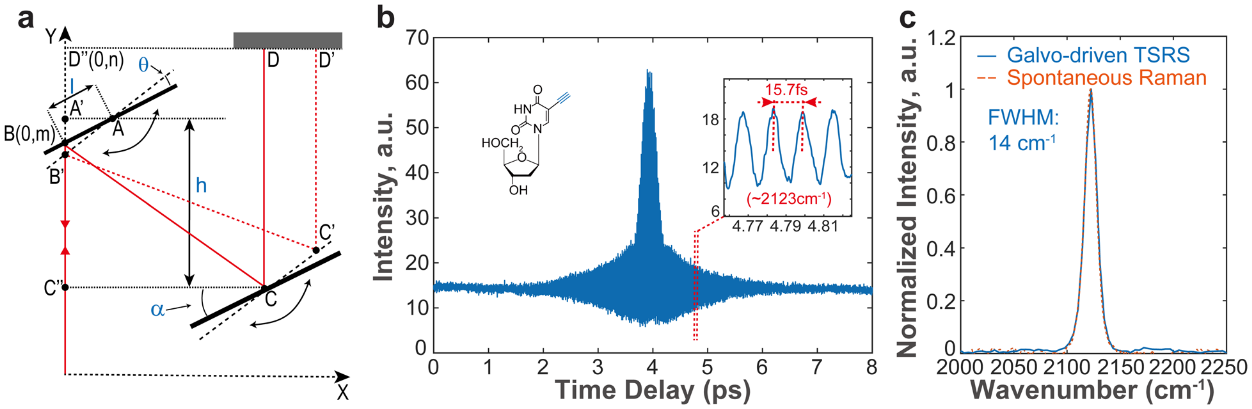

Fig. S6 All-plane-mirror high-speed delay scanning system. (a) The sketch of optical path during delay-scanning. A Cartesian coordinate system is built for convenience of calculation. (b) Time domain data of the C≡C mode of EdU acquired by linear-delay increments achieved by the rotation of galvanometer mirror pair. (c) The Fourier transform of time-domain data in (b). Orange dashed curve is spontaneous Raman spectrum of the C≡C mode of EdU measured by a commercial Raman spectrometer (0.65 cm^{-1} spectral resolution, LabRAM HR Evolution, Horiba) as a control.

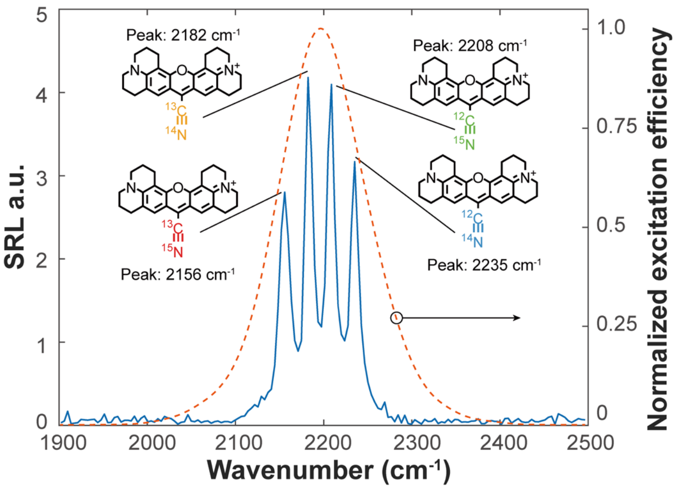

Fig. S7 Multiplexed T-SRS detection of the nitrile mode of Rhodamine 800 (Rh800). The sample is a 2.5-mM DMSO solution of the mixture of four Rh800 isotopologues (the nitrile bond is edited with 13C or 15N, none, or both). The red dashed curve shows the profile of excitation efficiency (i.e., the correlation of the laser spectra).

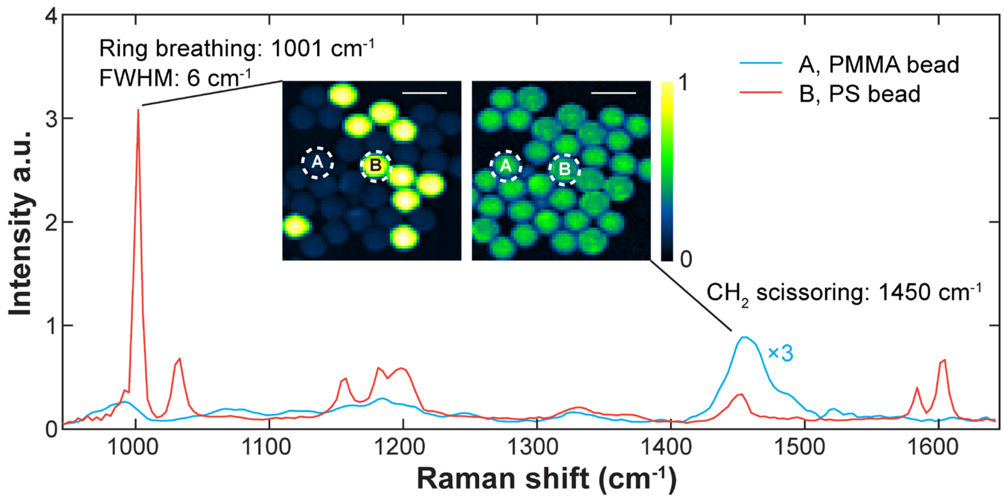

Fig. S8 T-SRS imaging of polymethyl methacrylate (PMMA) and polystyrene (PS) beads in the fingerprint region. The spectrum of the PMMA bead (labeled by 'A' in the inset panels) has been scaled by 3 times. Scale bar: 10 μm.

Details of data acquisitions

Our T-SRS system has two modes for data acquisition, depending on whether the delay-calibration data (the laser interference trace from the other output of the interferometer, see the grating and photodiode light path of the interferometer in **Fig. 3a**) is synchronously collected or not.

As mentioned in the above section (i.e., all-plane-mirror high-speed time-delay scanning), the two galvanometers for delay scanning can be driven with well-designed voltage signals (see **Fig. S4.a**) so that the time delay changes linearly. If the driven electronics of the galvanometers are well grounded and shielded, the accuracy of typical galvanometers used for laser microscopy enables natural-linewidth-limit spectral lines (as shown in **Fig. S6b,c**). In this condition, the motion errors can be omitted, and there is no need to record the delay-calibration data. We called this mode the free-run mode. Under this mode, the step size of delay scanning can be set up to 5 fs (i.e., the period of 3300 cm^{-1}, the upper limit of Raman spectrum in most cases, is ~ 10 fs). So, the 8-ps time delay range requires the sampling of at least 1600 data points for the time-domain SRL signal. Our data acquisition card (USB-6363, NI) enables a sample rate of up to 1 MHz when synchronic galvanometer driving and data acquisition are performed. So, the smallest dwell time for each pixel (or spectrum) is 1.6 ms for our current system. Note that the time-domain data near time-delay zero (i.e., the two pulse pairs are overlapping) should be removed to avoid aliasing of the laser spectra (i.e., undersampling of the laser interference fringes) to the Raman band. The details of such a time-gating trick have been discussed in the above section (i.e., non-resonant background subtraction for T-SRS).

To avoid instability induced by slight alignment changes of the interferometer or to be compatible with galvanometer systems that have relatively low accuracy, we provide the second data acquisition mode (i.e., data acquisition with the delay-calibration data synchronously collected) that guarantees the natural-linewidth-limit spectral lines. The delay-calibration data interference trace is the laser interference trace from the other output of the interferometer (generated by the interference of the 1030-nm frequency component, see the grating and photodiode light path of the interferometer in **Fig. 3a**). To ensure that the interference fringes are efficiently recorded, we have sampled more than ten points for every period (i.e., ~0.27 fs step size for delay sampling, one period of the 1030 nm interference fringe in the time-domain is ~3.4 fs, see **Fig. S9**). Under this mode, the 8-ps time delay range requires the sampling of 30,000 data points for both the time-

domain SRL signal and the delay-calibration data. Our data acquisition card (USB-6363, NI) enables the synchronic acquisition of the two signals at a sample rate of up to 1-MHz. So, the dwell time for every pixel in T-SRS images is 30 ms. Obviously, throughput is limited by the the sample rate of our data acquisition card under this calibrated mode. The current SNR we achieved suggest that improve the sampling rate by an order of magnitude is possible for most of the applications we have demonstrated.

As discussed above, the free-run mode enables much higher throughput, while the calibrated mode is more robust to motion errors and alignment drift in delay scanning, and more sampling points result in higher SNR. In this work, the delay scanning of our T-SRS imaging system is based on a pair of industry-level large-area galvanometers (S-9210, Sunny Technology) used for laser marking, which is much cheaper when compared with those standard products used for laser microscopy. To guarantee high accuracy, we have used the calibrated mode for data acquisition for all the images shown in **Fig.3-5**. Data in **Fig. S6b,c** is acquired in the free-run mode.

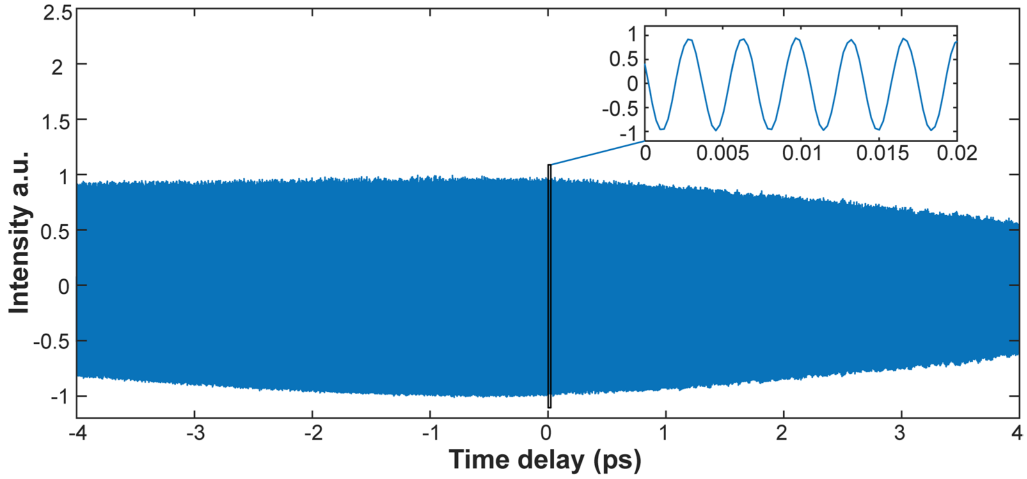

Fig. S9 A typical time-delay calibration signal synchronously recorded with the time-domain SRL signal. The step size is ~0.27 fs. The interference fringes of the narrowband laser components filtered out from the Stokes pulse (centered at 1030 nm) were used to track the delay scanning.

Supplementary Table 1. Probe concentrations (mM) used in fig.5a.

CAS # & name	Concentrations (mM)
**14235-81-5**<br>4-Ethynylaniline	172
**88444-81-9**<br>1-Ethynyl-3,5-bis(trifluoromethyl)benzene	594
**2170-06-1**<br>1-Phenyl-2-trimethylsilylacetylene	234
**17626-40-3**<br>3,4-Diaminobenzonitrile	64
**2920-38-9**<br>4-Phenylbenzonitrile	43
**98349-22-5**<br>2,4,5-Trifluorobenzonitrile	179
**1835-49-0**<br>Tetrafluoroterephthalonitrile	714

Supplementary Table 2. Probe concentrations (mM) for preparing barcoded PMMA beads.

CAS #	0110000	0001010	0100100	1100100	0001011	1010100	0001111
**14235-81-5 4-Ethynylaniline**	0	0	0	156	0	216	0
**88444-81-9 1-Ethynyl-3,5-bis(trifluoromethyl)benzene**	176	0	154	130	0	0	0
**2170-06-1 1-Phenyl-2-trimethylsilylacetylene**	94	0	0	0	0	41	0
**17626-40-3 3,4-Diaminobenzonitrile**	0	267	0	0	200	0	192
**2920-38-9 4-Phenylbenzonitrile**	0	0	7.7	6.5	0	6.8	0.52
**98349-22-5 2,4,5-Trifluorobenzonitrile**	0	67	0	0	67	0	87
**1835-49-0 Tetrafluoroterephthalonitrile**	0	0	0	0	67	0	105

References:

1 Berto, P., Andresen, E. R. & Rigneault, H. Background-free stimulated Raman spectroscopy and microscopy. *Physical review letters* **112**, 053905 (2014).

2 Xiong, H. *et al.* Background-free imaging of chemical bonds by a simple and robust frequency-modulated stimulated Raman scattering microscopy. *Opt. Express* **28**, 15663-15677, doi:10.1364/OE.391016 (2020).

3 Wallmeier, K. *et al.* Frequency modulation stimulated Raman scattering scheme for real-time background correction with a single light source. *Biomed. Opt. Express* **14**, 315-325 (2023).

4 Zhang, D., Slipchenko, M. N., Leaird, D. E., Weiner, A. M. & Cheng, J.-X. Spectrally modulated stimulated Raman scattering imaging with an angle-to-wavelength pulse shaper. *Opt. Express* **21**, 13864-13874 (2013).

5 Whaley-Mayda, L., Penwell, S. B. & Tokmakoff, A. Fluorescence encoded infrared spectroscopy: ultrafast vibrational spectroscopy on small ensembles of molecules in solution. *The journal of physical chemistry letters* **10**, 1967-1972 (2019).
